# Evaluation of an artificial intelligence-based clinical trial matching system in Chinese patients with hepatocellular carcinoma: a retrospective study

**DOI:** 10.1186/s12885-024-11959-7

**Published:** 2024-02-22

**Authors:** Kunyuan Wang, Hao Cui, Yun Zhu, Xiaoyun Hu, Chang Hong, Yabing Guo, Lingyao An, Qi Zhang, Li Liu

**Affiliations:** 1 State Key Laboratory of Organ Failure Research, Guangdong Provincial Key Laboratory of Viral Hepatitis Research, Department of Infectious Diseases and Hepatology Unit, Nanfang Hospital, Southern Medical University, No. 1838, North Guangzhou Avenue, Baiyun District, Guangzhou, China; 2 Research and Development Department, Huimei Technology Co., Ltd, Beijing, China; 3 Big Data Centre, Nanfang Hospital, Southern Medical University, Guangzhou, China

**Keywords:** Clinical trial matching, Artificial intelligence, Screening, Machine learning, Hepatocellular carcinoma

## Abstract

**Background:**

Artificial intelligence (AI)-assisted clinical trial screening is a promising prospect, although previous matching systems were developed in English, and relevant studies have only been conducted in Western countries. Therefore, we evaluated an AI-based clinical trial matching system (CTMS) that extracts medical data from the electronic health record system and matches them to clinical trials automatically.

**Methods:**

This study included 1,053 consecutive inpatients primarily diagnosed with hepatocellular carcinoma who were referred to the liver tumor center of an academic medical center in China between January and December 2019. The eligibility criteria extracted from two clinical trials, patient attributes, and gold standard were decided manually. We evaluated the performance of the CTMS against the established gold standard by measuring the accuracy, sensitivity, specificity, positive predictive value (PPV), negative predictive value (NPV), and run time required.

**Results:**

The manual reviewers demonstrated acceptable interrater reliability (Cohen’s kappa 0.65–0.88). The performance results for the CTMS were as follows: accuracy, 92.9–98.0%; sensitivity, 51.9–83.5%; specificity, 99.0–99.1%; PPV, 75.7–85.1%; and NPV, 97.4–98.9%. The time required for eligibility determination by the CTMS and manual reviewers was 2 and 150 h, respectively.

**Conclusions:**

We found that the CTMS is particularly reliable in excluding ineligible patients in a significantly reduced amount of time. The CTMS excluded ineligible patients for clinical trials with good performance, reducing 98.7% of the work time. Thus, such AI-based systems with natural language processing and machine learning have potential utility in Chinese clinical trials.

**Supplementary Information:**

The online version contains supplementary material available at 10.1186/s12885-024-11959-7.

## Background

Clinical trials are critical in cancer research when introducing advanced therapies or devices into clinical practice as their overarching goal is reducing cancer mortality and prolonging patient survival [1]. Patients with cancer are generally recommended to participate in clinical trials; however, a large proportion of patients have no access to screening and enrollment. In contrast, 20–40% of cancer clinical trials are impeded or fail because of numerous reasons, including the lack of candidates [1–6]. The routine clinical work burden of care providers, time limitations of skilled staff, and difficulties in analyzing substantial information contribute to this issue [7–9]. Owing to the increasing quantity and complexity of clinical trials [10, 11], additional effort is required in the screening process and in matching numerous patients to one possible trial or several active trials to one patient.

Screening patients and identifying those who meet the eligibility criteria is often a knowledge-demanding and time-consuming task [8, 9, 12, 13]. Therefore, the steps to screen patients rapidly and accurately have become a matter of concern. The development of health information technologies using artificial intelligence (AI), such as natural language processing (NLP) and machine learning (ML), provides a potential means of screening patients automatically. AI-based technology may enhance screening efficiency and accuracy, reduce research team fatigue, and improve the clinical trial accrual consequently [3, 8, 12]. In 2015, Ni et al. reported on an automated clinical trial eligibility screening approach in an emergency department [14]. In 2020, another automatic matching system for clinical trials, termed Mendel AI, was tested [15]. IBM Corp.’s Watson is another good AI tool for clinical trial matching (CTM) that has been tested in different countries and cancer types. This software platform can be integrated with the local electronic health record (EHR) system and identify patients as “Exclude” or “Consider” for clinical trials, based on the interpretation of trial protocols, patient information retrieved from EHRs or databases, and full- or semi-automatically decided attributes [3, 8]. A promising prospect has been reported in AI-assisted clinical trial screening [16, 17]; nevertheless, the matching systems have been developed in an English language environment, and relevant studies have been conducted only in Western countries. China’s clinical records often comprise substantial descriptive content of medical history rather than structured information, and the semantic recognition of Chinese sentences is different to that of English [18]. Hence, our team of oncologists and computer specialists have developed a clinical trial matching system (CTMS), which is integrated with the Chinese EHR system. We aimed to evaluate CTMS against two clinical trials for a cohort of hospitalized patients with hepatocellular carcinoma (HCC) retroactively.

## Methods

### Patients

This study included 1,053 consecutive inpatients who were referred to the liver tumor center of Nanfang Hospital, Southern Medical University, Guangzhou, China, from January 2019 to December 2019. The patients were primarily diagnosed with HCC before admission. This cohort consisted of heterogeneous samples, such as new patients, previously treated patients, clinical trial enrollees, and patients with non-HCC liver tumors.

### Data collection

The eligibility criteria were extracted manually from two clinical trials conducted at our institution in 2019 and clarified by two oncologists. Trial No. 1 was a phase III first-line drug research for advanced HCC (NCT04194775) and Trial No. 2 was a non-inferior study of a premarketing ablation device for untreated early HCC (a trial authorized by Provincial Medical Products Administration). Thus, the potential candidates were not competitive. The original eligibility criteria were not adopted completely for CTMS matching because some of them were highly dependent on the clinician’s subjective judgment, such as an expected patient survival of > 3 months, and some could be “awaited,” that is at least a 2-week interval after an intervention. The patient attributes were decided manually by two senior oncologists and computer specialists.

A gold standard for trial eligibility was determined for each patient against the two clinical trials by two senior oncologists by manually reviewing all information in EHRs (examination images included). The oncologists screened the patients against the original trial protocols and made subjective judgments when necessary. Another senior oncologist reviewed the results with discrepancies to achieve consensus. The time required for manual review was recorded.

We attempted to assess the NLP and ML capabilities of the CTMS without separation. Clinical data on the included patients were extracted from local EHRs without manual input, and identifiable information was redacted. The CTMS was originally designed to provide instant eligibility advice via the EHR reminder; however, it was specially adjusted because of the retrospective study design. The historical interventions (anti-tumor treatments and clinical trial enrollment) received by a patient were analyzed as medical history; nonetheless, any new intervention administered during the hospitalization was automatically ignored, simulating a scenario in which the patient met the eligibility criteria upon not receiving the latest interventions.

Clinical data included both structured and unstructured data. Structured data consisted of demographic variables, such as sex, age, height, weight, and laboratory results, whereas unstructured data sources were defined as clinical notes and examination reports. Each patient was assessed and classified as “Exclude” (not eligible) or “Consider” (potentially eligible) by the CTMS. The reliability of exclusion was prioritized because we assumed the primary task of the CTMS was to reduce the tedious work of exclusion, considering the general capabilities of AI.

### CTMS architecture

**Fig. 1:**
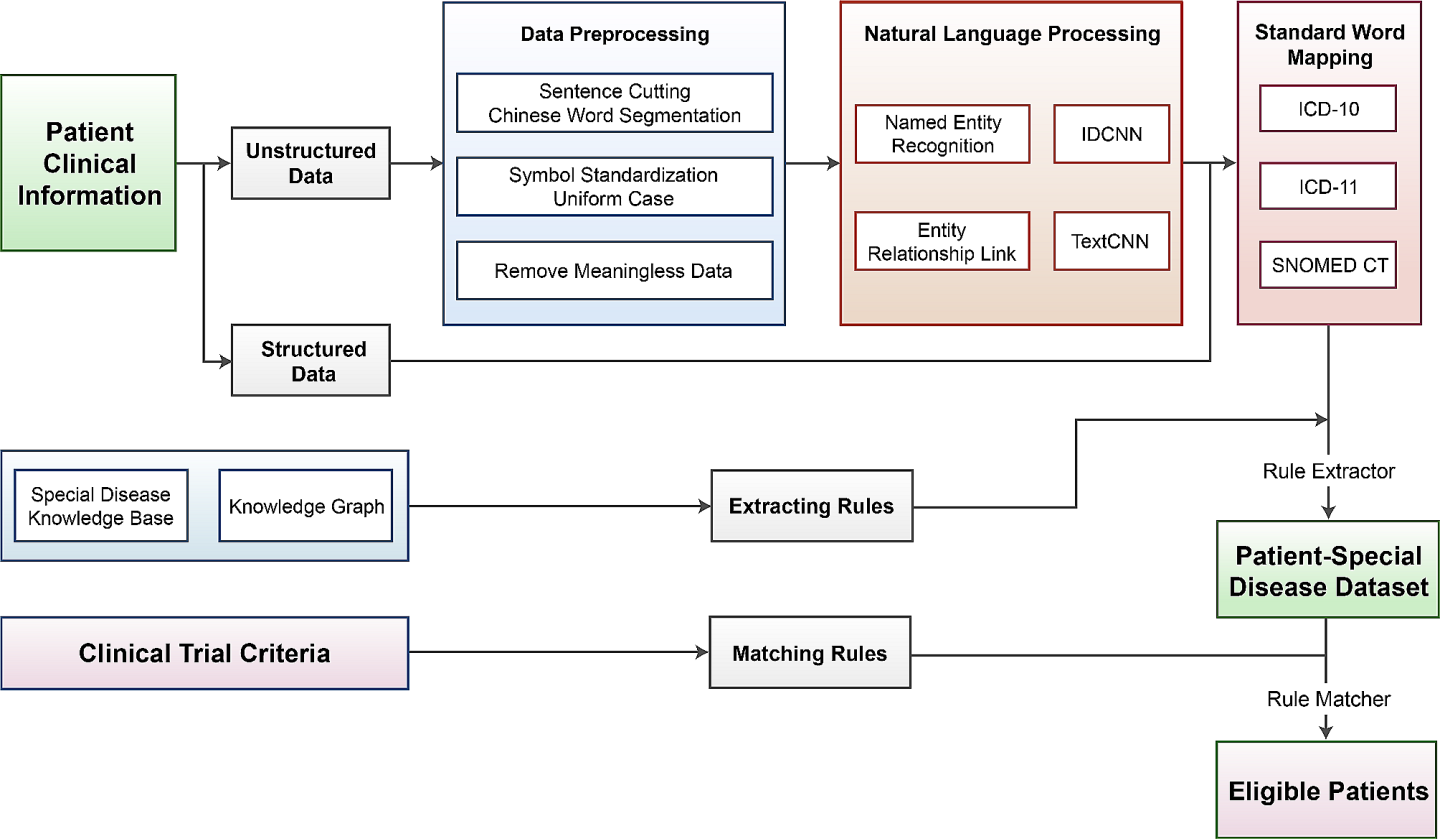
Framework of clinical trial matching system (CTMS). Abbreviations: TextCNN, text convolutional neural networks; IDCNN, dilated convolutional neural networks; ICD, international classification of diseases; and SNOMED CT, Systematized Nomenclature of Medicine-Clinical Terms

The CTMS framework (Fig. 1) consisted of three primary steps as follows: the extraction of medical data, construction of patient-specific disease datasets, and matching patients. After NLP based data preprocessing that included sentence cutting, Chinese word segmentation, symbol standardization, and removal of meaningless data, unstructured clinical data of patients were post-structured. We used the named entity recognition (NER) model of the iterated dilated convolutional neural networks (IDCNN) architecture to extract medical entity words, such as time, location, anatomical site, diagnosis, tumor stage, laboratory test, drug name, drug dosage, surgery name, and presence status. The entity-relationship link model of the text convolutional neural networks (TextCNN) extracted the medical entity combination relationship, such as the diagnosis-anatomical part-orientation-time as a group. Subsequently, core entities, such as diagnosis, laboratory examination, and drug name, were mapped to the medical standard words (ICD-10, ICD-11 and SNOMED CT), and the attributes, such as the time, existence state, and drug dosage, were attached to the medical standard words. The approximately 20,000 medical data sets for training of the NER and TextCNN model primarily originated from the competition data of Chinese Biomedical Language Understanding Evaluation (CBLUE) 2.0 [https://tianchi.aliyun.com/cblue]. The CBLUE was composed of the previous academic evaluation competitions of the China health information process conference and the data sets of the Aliquark medical search business, including medical text information extraction, term standardization, text classification, sentence semantic relationship determination, and dialogue understanding and generation, including 14 sub-tasks in five categories. We constructed a patient-special disease dataset through the following steps: a special disease knowledge base for HCC, including approximately 150,000 data sets derived from guidelines, literature, and books was developed. Leveraging the knowledge graph technology, we analyzed this knowledge base to derive extracting rules that specified the location, format, and content type our model should extract from medical records. Subsequently, the rule extractor filtered the outcomes of standard word mapping to generate the patient-special disease dataset which included primary modules, sub-modules, data elements, and value ranges, etc. Finally, computer experts and oncologists divided and optimized the two clinical trial criteria into computer-implemented criteria, which were matching rules. Then the rule matcher selected eligible patients from the patient-special disease dataset.

### Statistical analysis

Interrater reliability between the clinicians involved in the manual review leading to a consensus on gold standard was measured by Cohen’s kappa with reported standard error (SPSS 25.0; IBM Corp., Armonk, NY, USA). We measured the accuracy, sensitivity, specificity, positive predictive value (PPV), and negative predictive value (NPV) of the CTMS against the established gold standard as follows: accuracy (agreement) = (TP + TN) / (TP + FN + FP + TN); sensitivity (recall) = TP / (TP + FN); specificity = TN / (TN + FP); PPV (precision) = TP/ (TP + FP); NPV = TN / (TN + FN), where TP is defined as true positive, FN as false negative, TN as true negative, and FP as false positive.

## Results

We assessed 1,053 patients with 3,064 hospitalization records in 2019 for eligibility against the two clinical trials. Table 1 summarizes some of the patient attributes derived by the CTMS. The CTMS supplemented the absent items that could not be extracted directly from the EHRs, such as tumor status, using NLP. Supplementary Tables S1 and S2 describe the two clinical trials and eligibility criteria [see Additional file 1].

**Table 1:** Part of the patient attributes derived from CTMS

Attribute	No.	%
Age ≥ 18 years	1,048	99.5
Diagnosis		
HCC	889	84.4
Non-HCC	164	15.6
Tumor status		
BCLC B or C stage	686	65.1
Progressed and measurable lesions^a^	228	21.7
Diffused or massive intrahepatic lesions	50	4.7
Diameter > 3 cm	553	52.5
Tumor adjacent to main vessels or gallbladder	64	6.1
Tumor thrombosis in main trunk of PV or in IVC	150	14.2
CNS metastases	12	1.1
ECOG-PS > 2	54	5.1
Child-Pugh ≥ 10	44	4.2
Large volume of ascites	51	4.8
Serious non-healing wound or fracture	2	0.2
Laboratory results		
HBV-DNA > 2,000 IU/mL	70	6.6
ALT or AST > 5 ULN	290	27.5
Medical history		
Current participation in clinical trials	84	8.0
Prior systemic anti-tumor treatments	493	46.8
HE	41	3.9
AIDS	6	0.6
Major cardiovascular diseases	11	1.0
Immune diseases underwent steroid treatment	16	1.5
Tuberculosis	20	1.9
Transplantation	10	0.9

^a^Note: the definition of progressed and measurable lesion was consistent with the Response Evaluation Criteria in Solid Tumours (version 1.1). Abbreviations: HCC, hepatocellular carcinoma; BCLC, Barcelona Clinic Liver Cancer; PV, portal vein; IVC, inferior vena cava; CNS, central nervous system; ECOG-PS, Eastern Cooperative Oncology Group Performance Status; HBV, hepatitis B virus; ALT, alanine aminotransferase; AST, aspartate aminotransferase; ULN, upper limit of normal; AIDS, acquired immune deficiency syndrome; and HE, hepatic encephalopathy

The CTMS evaluated 171,584 patient attributes against 113,368 individual eligibility criteria for trial No. 1 and 58,216 attributes against 64,344 individual eligibility criteria for trial No. 2. The median time for the CTMS to run a query and perform matching for each hospitalization record was 577 ms (range 145–828) and 511 ms (range 130–746) for trial Nos. 1 and 2, respectively. The overall time required was 60 min and 53 min for trial Nos. 1 and 2, respectively. Conversely, each skilled oncologist took approximately 150 h to screen all the patients manually against the two trials simultaneously.

The interrater reliability was acceptable while establishing the gold standard (Cohen’s kappa 0.65–0.88, Table 2). The disagreement consisted of overlooked patient characteristics or discrepant subjective judgment, such as evaluating expected patient survival or suitability for ablation.

**Table 2:** Interrater reliability between manual reviewers

Trial No.	Kappa	Standard Error	P value	Agreement
1	0.653	0.043	< 0.001	94.49
2	0.881	0.026	< 0.001	98.10

For trial No. 1, the CTMS identified 37 “Consider” patients and 1,016 “Exclude” patients, with 92.9% accuracy, 51.9% sensitivity, 99.1% specificity, 75.7% PPV, and 97.4% NPV. For trial No. 2, the CTMS identified 67 “Consider” patients and 986 “Exclude” patients, with 98.0% accuracy, 83.5% sensitivity, 99.0% specificity, 85.1% PPV, and 98.9% NPV (Table 3). The performance of the CTMS on trial No. 1 was inferior to that on trial No. 2 mainly owing to lower sensitivity. Notably, the actual number of participants included in the two clinical trials in 2019 was 19 and 39, respectively; all of these participants were classified as “Consider” by the CTMS.

**Table 3:** Performance of CTMS matching of 1,053 patients against two trials

Trial No.	Consider^a^ (No.)		Exclude^a^ (No.)		Accuracy (%)	Sensitivity (%)	Specificity (%)	PPV (%)	NPV (%)
	TP	FP	TN	FN					
1	37		1016		92.9	51.9	99.1	75.7	97.4
	28	9	990	26					
2	67		986		98.0	83.5	99.0	85.1	98.9
	57	10	975	11					

^a^Note: CTMS classified patients as “Consider” for potential eligibility and “Exclude” for ineligibility. Abbreviations: CTMS, clinical trial matching system; TP, true positive; FP, false positive; TN, true negative; FN, false negative; PPV, positive predictive value; and NPV, negative predictive value

Table 4 summarizes the misclassification by the CTMS. Some errors were caused by the insufficient capability of the CTMS, and the remaining were caused by the insufficient quality of the medical records and discrepancies between the CTMS and manual reviewers’ subjective judgment.

**Table 4:** CTMS misclassification and its details

Misclassification category	Misclassification details	No. of misclassifications per trial				
		No. 1		No. 2		All
		FP	FN	FP	FN	
Data collection	Collect data inaccurately because of inadequate integration with EHRs	7		12		19
		1	6	3	9	
Misjudgment	Misjudgments due to inadequate capability of CTMS or poor medical record qualification	25		9		34
		5	20	7	2	
Criteria unadopted	Reviewers excluded some patients based on the eligibility criteria that were not adopted by CTMS, such as an expected patient survival	3		0		3
		3	0	0	0	

Abbreviations: CTMS, clinical trial matching system; EHR, electronic health record; FP, false positive; and FN, false negative

## Discussion

In this study, we assessed the performance of the CTMS against two clinical trials in patients with both early- and advanced-stage HCC. We observed a high specificity and good sensitivity. Generally, the results were not inferior to those of previous studies. In 2019, Australian researchers evaluated a CTM system using Watson in 102 patients with lung cancer, revealing 91.6% accuracy (vs. 92.9–98.0% in the current study), 83.3% sensitivity (vs. 51.9–83.8%), 93.8% specificity (vs. 99.0–99.1%), 76.5% PPV (vs. 75.7–85.1%), and 95.7% NPV (vs. 97.4–98.9%) [8]. In another study conducted by the Highlands Oncology Group in 2019, 239 patients with breast cancer were assessed for eligibility by Watson against four clinical trials [3], and their eligibility determination was reported with 81–96% agreement, 76–99% specificity, and 91–95% sensitivity for three trials and 46.7% for the fourth trial. In another study conducted at the Mayo Clinic, Watson was used to evaluate 318 patients with breast cancer against four clinical trials. The interrater reliability for manual assignment demonstrated a 0.60 to 0.77 Cohen’s kappa and an accuracy of 87.6%, with 81.1% sensitivity and 89% specificity [12]. Notably, in our study, the CTMS took considerably less time than manual work for screening, reducing the time required by 98.7% (2 h vs. 150 h). The manual reviewers inevitably had some advantages reviewing the non-anonymous EHRs, such as the exposure to actual enrollment information in medical records. The outcome of our study demonstrated that the AI-based matching system could help screen patients with cancers other than those of breast and lung accurately and efficiently; nonetheless, manual review against the “Consider” patients remains indispensable.

Our study had some strengths. First, this novel study developed a CTMS applicable to Chinese language and evaluated its performance in a real-world HCC cohort. Clinical data have been widely extracted from EHRs using NLP in English language [8, 12]. However, it is challenging in Chinese because the semantic recognition is highly dependent on the phrase and word group. Furthermore, cross-ambiguity and combinatorial ambiguity occur more easily [19]. Second, IDCNN was used for NER in the CTMS [20]. Compared with the traditional convolution networks, IDCNN can obtain a larger receptive field for the convolution kernel of a similar size by introducing the dilation rate, reduce the problem of information loss caused by the size limitation of the convolution kernel, and obtain a higher accuracy of entity recognition. Using lightweight TextCNN in the entity-relationship link can ensure the high accuracy of entity-relationship recognition and achieve a faster reasoning speed [21]. Third, to simulate a practical process of screening in manual reviewing, we allowed the experts to review the EHRs (images included) and exercise their subjective judgment completely. In the Australia study, clinical data were extracted from a prospective observational cohort study database and entered into CTM automatically and manually. While establishing the gold standard, the reviewers determined the eligibility by reviewing the patient attributes entered in CTM rather than the complete medical records because of the lack of integrated EHRs. Thus, the ability of NLP may not be assessed sufficiently. Fourth, the HCC trials had complicated eligibility criteria, and a large amount of data from the 1,053 patients should be processed by the CTMS during the 1-year study period. In comparison, only one medical record of each patient (without pathology records) during a 16-week study period was used for attribute extraction and eligibility determination in the study of the Highlands Oncology Group, and a manual review of each patient was performed by only one trial coordinator.

In the future, systems such as CTMS can be used as an investigation tool before selecting a clinical trial site. The CTMS can evaluate the feasibility and identify the time needed to enroll a sufficient number of participants in trials using local medical data retrospectively. This procedure will be more accurate and efficient than the current method of using questionnaires. Moreover, the potential application of such systems could help researchers establish a network of candidate recommendations between hospitals, accelerating research progress. However, the capabilities of such systems require further optimization, and prospective studies are needed to confirm its advantages in clinical practice [22].

This study had several limitations. First, it was a single-center, retrospective study comprising only two clinical trials because of the manual workload. Second, 93.5–94.9% of patients were ineligible for the trials owing to the rigorous criteria of clinical trials and the nature of the unselected patient group. The system performed well in exclusion with a 97.4–98.9% NPV and a 99.0–99.1% specificity; however, the number of FN was inevitably close to that of TP, and the outcome of sensitivity was consequently affected. The unsatisfactory sensitivity in our findings can be attributed to this, as well as the insufficient capability of CTMS. Finally, we did not evaluate the CTMS NLP and ML capabilities separately, and the adoption of criteria and the determination of attributes required manual work.

## Conclusions

Our findings preliminarily demonstrated that the CTMS can integrate with the Chinese EHR system, retrieve clinical data automatically, and match patients to clinical trials for early and advanced HCC. Notably, the CTMS is particularly reliable in excluding ineligible patients in a significantly reduced amount of time. Additional prospective studies are still required to evaluate the impact of such AI-based systems on real-world clinical trial accrual.

### Electronic supplementary material

Below is the link to the electronic supplementary material.

Supplementary Material 1

## Data Availability

All data generated or analyzed during this study are included in this published article and its supplementary information files.

## Abbreviations

AI: Artificial intelligence
CBLUE: Chinese Biomedical Language Understanding Evaluation
CTM: Clinical trial matching
CTMS: Clinical trial matching system
EHR: Electronic health record
FN: False negative
FP: False positive
HCC: Hepatocellular carcinoma
ICD-10: The International Statistical Classification of Diseases and Related Health Problems 10th Revision
ICD-11: International Classification of Diseases 11th Revision
IDCNN: Iterated dilated convolutional neural networks
ML: Machine learning
NER: Named entity recognition
NLP: Natural language processing
NPV: Negative predictive value
PPV: Positive predictive value
SNOMED CT: Systematized Nomenclature of Medicine-Clinical Terms
TextCNN: Text convolutional neural networks
TN: True negative
TP: True positive
