# Supplementary material for: Evaluation of an artificial intelligence-based clinical trial matching system in Chinese patients with hepatocellular carcinoma: a retrospective study
Source: BMC Cancer. 2024 Feb 22;24:246. doi: 10.1186/s12885-024-11959-7 (PMC10885498; doi:10.1186/s12885-024-11959-7)
Supplement: Supplementary file 1 — Supplementary Material 1 [file 12885_2024_11959_MOESM1_ESM.docx]

## Supplementary Table 1. Clinical trial features

| **Trial No.** | **Phase** | **Design** | **Intervention** | **Population** | **Registration or authorization** |
| --- | --- | --- | --- | --- | --- |
| 1 | 3 | 2-arm RCT | PD-1 inhibitor + targeted drug versus placebo + targeted drug | Advanced HCC without prior systemic therapy | NCT04194775 |
| 2 | 3 | 2-arm RCT | Premarketing microwave ablation device versus microwave ablation device on the market | Early HCC with untreated lesions | Jiangsu Provincial Medical Products Administration (20180917) |

**Abbreviations**: RCT, randomized controlled trial; PD-1, programmed death-1; and HCC, hepatocellular carcinoma

## Supplementary Table 2. Examples of eligibility criteria

|  | Original protocol criteria | Clarified or refined criteria for CTMS |
| --- | --- | --- |
| Inclusion criteria | Patients with unresectable advanced HCC that is not eligible for surgery and/or locoregional therapy (Stage B or C based on the Barcelona Clinic Liver Cancer [BCLC] staging system) and meets either one of the following criteria: 1) histologically or cytologically confirmed diagnosis of HCC; 2) clinically confirmed diagnosis of HCC according to the American Association for the study of Liver Diseases (AASLD) criteria. Patients without cirrhosis require a histological confirmation of diagnosis. The curative treatment and/or locoregional therapy must have been completed ≥4 weeks before the baseline scan for those who experienced disease progression. | Patients diagnosed with HCC, BCLC B, or C stage. |
|  | At least one measurable lesion can be assessed by the investigator based on the Response Evaluation Criteria in Solid Tumors (RECIST) v1.1 within 14 days before randomization. Target lesions in the past radiation fields or that underwent locoregional therapy (e.g., TACE or ablation), if confirmed as radiographic progression, are considered as measurable lesions. | At least one measurable lesion can be assessed based on the Response Evaluation Criteria in Solid Tumors (RECIST) v1.1. Target lesions in the past radiation fields or that underwent locoregional therapy, if confirmed as radiographic progression, are considered as measurable lesions. |
|  | Liver function of Child-Pugh A or B class or meet this standard after treatment. | Liver function of Child-Pugh A or B class |
| Exclusion criteria | A prior bleeding event caused by esophageal or gastric varices within 6 months or other gastrointestinal bleeding events within 28 days before screening. Untreated or incompletely treated esophageal or gastric varices that are considered by the investigator to be at high-risk for bleeding (Note: Patients must undergo an esophagogastroduodenoscopy [EGD], and all size of varices [small to large] must be assessed and treated per the local standard of care before enrollment; patients who have undergone an EGD within 6 months before the initiation of study treatment do not need to repeat the procedure). Active gastric or duodenal ulcer. | A prior bleeding event caused by esophageal or gastric varices within 6 months. |
|  | HBV and HCV co-infection (Note: Subject has HCV infection history, but HCV RNA is undetectable at screening can be considered as non-HCV infection in this study). | HBV infection and HCV-RNA positive |
|  | Major cardiovascular diseases (e.g., congestive heart failure, unstable angina, atrial fibrillation, and severe arrhythmia); any of acute myocardial infarction, unstable angina, stroke, or transient ischemic attack within 6 months before enrollment; New York Heart Association (NYHA) grade ≥2 congestive heart failure. | Major cardiovascular diseases or NYHA grade ≥2 congestive heart failure |
|  | Tumor adjacent to primary vessels | Tumor adjacent to portal vein and its branches, or hepatic veins, or inferior vena cava |

Abbreviations: CTMS, clinical trial matching system; TACE, transarterial chemoembolization; HBV, hepatitis B virus; and HCV, hepatitis C virus
